# Review of Real-Time Biomechanical Feedback Systems in Sport and Rehabilitation

**DOI:** 10.3390/s22083006

**Published:** 2022-04-14

**Authors:** Matevž Hribernik, Anton Umek, Sašo Tomažič, Anton Kos

**Affiliations:** Faculty of Electrical Engineering, University of Ljubljana, 1000 Ljubljana, Slovenia; matevz.hribernik@fe.uni-lj.si (M.H.); anton.umek@fe.uni-lj.si (A.U.); saso.tomazic@fe.uni-lj.si (S.T.)

**Keywords:** real-time, concurrent, instant, biomechanical feedback, system, application, sensor, actuator, modality, motion, sport, rehabilitation

## Abstract

Real-time biomechanical feedback (BMF) is a relatively new area of research. The potential of using advanced technology to improve motion skills in sport and accelerate physical rehabilitation has been demonstrated in a number of studies. This paper provides a literature review of BMF systems in sports and rehabilitation. Our motivation was to examine the history of the field to capture its evolution over time, particularly how technologies are used and implemented in BMF systems, and to identify the most recent studies showing novel solutions and remarkable implementations. We searched for papers in three research databases: Scopus, Web of Science, and PubMed. The initial search yielded 1167 unique papers. After a rigorous and challenging exclusion process, 144 papers were eventually included in this report. We focused on papers describing applications and systems that implement a complete real-time feedback loop, which must include the use of sensors, real-time processing, and concurrent feedback. A number of research questions were raised, and the papers were studied and evaluated accordingly. We identified different types of physical activities, sensors, modalities, actuators, communications, settings and end users. A subset of the included papers, showing the most perspectives, was reviewed in depth to highlight and present their innovative research approaches and techniques. Real-time BMF has great potential in many areas. In recent years, sensors have been the main focus of these studies, but new types of processing devices, methods, and algorithms, actuators, and communication technologies and protocols will be explored in more depth in the future. This paper presents a broad insight into the field of BMF.

## 1. Introduction

The use of smart devices and wearable technology in everyday life has increased considerably in the last decade [1,2,3,4]. This is also true in the field of sports and physical rehabilitation. In sports, professional and recreational athletes are constantly trying to improve their skills and performance by using advanced (wearable) technology, which can ultimately lead to a competitive advantage [5,6]. Similarly, in physical rehabilitation, patients use technology to shorten the rehabilitation process and improve its effectiveness [7].

A large number of electronic devices used in sports and physical rehabilitation are classified into three groups using terminology in the field of electronics: consumer devices, high-end devices and systems and purpose-built devices and systems. *Consumer devices* include smart wristbands, heart rate monitors, smartwatches, smartphones, and many others. They usually contain various sensors, such as kinematic sensors to measure motion [8] and sensors to measure physiological parameters of the user. Generally, users employ these devices to self-quantify their physical activity. Consumer devices are easy to use and predominantly provide some sort of statistical results, such as event counts, averages, progress, and the like. *High-end devices and systems* are primarily used for professional laboratory testing and research. They include optical tracking systems, force plate systems, video analysis systems, and others. They provide precise and accurate measurements of various activity parameters, such as movement patterns or force distribution, but their use is limited and restricted to specific tasks, environments and experts; not to mention their high acquisition and operating costs. *Purpose-built devices and systems* attempt to bridge the gap between consumer and high-end devices and systems. In most cases, they are designed to be task-specific and implemented (at least in part) as wearable devices. They are used to assess the kinetics and kinematics of movement or physiological parameters of the user, preferably without interfering with the performance of the activity. In general, their use is more complex than consumer devices, which typically do not require special setup, but less complex than high-end systems, which may require complicated setup and trained personnel. Purpose-built devices and systems using wearable technology enable task-specific acquisition of biomechanical and physiological parameters of activity. When compared to consumer or high-end devices, the task-specific data allows for targeted processing and analysis, resulting in higher quality results regarding the specific task. This ultimately increases rehabilitation benefits and training performance [9,10,11].

**Figure 1:**
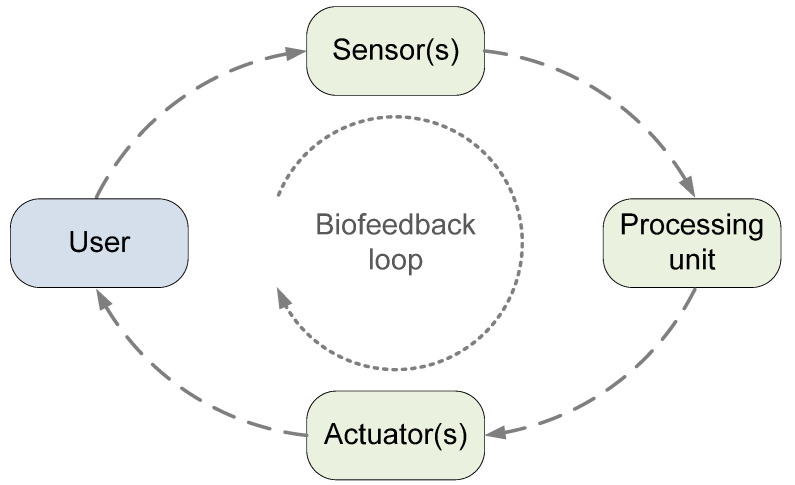
BMF system—Sensor(s) measure the user’s activity and send data to the processing unit. The processing unit analyses the data and generates feedback that is provided to the user via the actuator(s). The user responds to this feedback by correcting or changing their activity.

One concept that leads to improved execution of activity and performance, and thus a competitive advantage in sports or a better rehabilitation process, is **biomechanical feedback (BMF) [2,5,6,7]**. BMF is a treatment and body control method that uses sensors to measure a person’s physiological and physical body functions, parameters, and activities that cannot be perceived by the human senses. The data from the sensors are processed and the results are fed back to the person via one of the human senses. The person tries to respond to the received information in order to change the sensed functions, parameters and activities in the desired way, thus closing a feedback loop [2]. BMF systems generally consist of at least four basic elements: a user, one or more sensors, a processing unit, and one or more actuators, as shown in Figure 1. There can be more than one instance of each element [2].

Different *sensors* that measure physiological or biomechanical parameters of the body [12] are used and studied in different applications in sports and physical rehabilitation [13]. The selection of the most suitable sensors depends strongly on the measured parameters and variables, on the activity performed and on the intended use of the BMF system. 

The *processing unit* receives signals and data from sensors and processes them. The type and operation of the processing unit depends on the processing requirements of the system. The sensor and the processing unit may be integrated into the same device or they may be implemented as two separate devices. In the latter case, they are usually wirelessly connected, but in certain cases a wired connection is also used. Theoretically, there is no limit to the number of processing units; multiple computers may share the task of processing data, but this is usually not required. Some or all processing tasks can also be performed in the cloud. Currently, there is no need for devices with high processing power, and, in general, processing power is not an issue in application development, as microcontrollers and personal computers can perform almost all required tasks.

Feedback information must be meaningful and understandable to the user. It is provided in different modalities defined by stimulation of the human senses: auditory for sound, visual for sight, vibrotactile or haptic for touch. The BMF system communicates with the user by presenting information through a variety of *actuators* such as visual displays, headphones or speakers, vibrating wristbands, or augmented reality glasses. It is very important to use the right type of modality—the feedback information should not interfere with modalities used to perform the activity and should not impose too much additional cognitive load. Nevertheless, different modalities can be used simultaneously or in the same application, but only under the conditions mentioned above.

In BMF systems, it is very important how early we provide the feedback information to the user in relation to the activity being performed. We understand the concept of timing from two perspectives: the user’s perspective and the system’s perspective. Users experience feedback in different modes: concurrent, cyclic, terminal, and event-driven. *Concurrent feedback* is provided throughout the duration of the activity. *Cyclic feedback* is given after completion of a specific part or cycle of an activity. *Event-driven feedback* is given to the user only at times when certain events (triggers) occur during the movement and when these events are significant to the user. *Terminal feedback* is given after the action. Feedback timing, from the perspectives of the system, can be categorized into real time or post processing feedback. The understanding of what is real time varies in different domains. In BMF systems the notion of real-time depends on human perception of the operation of the system. The BMF system interacts with the user via feedback modality, and the user interacts with the system by changing motion that is detected by sensors. We can assume that a system operates in real time, if the user is not able to perceive the interaction as delayed or uncoordinated [14,15]. Depending on the stimulus (feedback modality), humans can normally perceive latencies between 25 ms (auditory and haptic) and 100 ms (visual) [14]. The total delay of the BMF loop includes the sum of delays of all BMF system elements and the communication between them. We can conclude that *real-time BMF* systems should operate under the above constraints to be effective and perceived as natural. Unlike real-time feedback, *post-processing feedback* is much easier to implement because there are no strict and rigid time constraints on when processing must be carried out. This review focuses only on real-time BMF systems.

The implementation of real-time BMF systems and applications may vary in different sports or rehabilitation cases. A BMF system for aquatics sports may be quite different from a system for martial arts or a system for gait rehabilitation. However, similar or even the same systems can also be used in both sport and rehabilitation. For example, the same BMF system can be used in running practice and in gait rehabilitation. Additionally, Wi-Fi communication and smartphone display actuators are applicable in many cases in sports, but they are useless under water. When designing BMF devices, obstructiveness is an important factor that limits the size, weight, type of communication, and operation of the device. The device should not restrict the user, should not obstruct the movement, and should be virtually imperceptible.

Sensors are often integrated into wearable sensor devices that contain a processor (i.e., a microcontroller), a battery, and a (wireless) communication module, allowing the sensor device to communicate with other devices such as computers, smartphones, or tablets. Wearable sensor devices must be small, lightweight and should have sufficiently long autonomy. Signal and data processing and analysis is performed on a wearable device itself or sent to a computer or smart device. Digital signal processing techniques are used for analysis and feedback generation. The actuator must also communicate with the processing unit. It can use the same communication technology as the sensor or a different one. The choice of actuator depends on the modality used. Auditory feedback can be provided to the athlete through headphones, speakers, or other acoustic devices. Visual feedback can be displayed on a screen or projector, via smart glasses or a VR/AR (virtual reality/augmented reality) headset. Vibrotactile feedback is typically generated with small haptic vibrators or piezoelectric elements. In some implementations, the sensor, processing unit and actuator are integrated into the same device, such as smartwatches or smartphones.

The main motivation for this review was our long-term involvement and work in the development and implementation of sensor technologies and BMF systems in sports. Our primary objective was to examine how different research groups use available technologies to develop and implement BMF systems. Therefore, the review concentrates primarily on the technological aspects of BMF rather than other aspects in sports and rehabilitation field, such as software applications and user-system interactions. In this review, we focus only on real-time BMF systems that provide concurrent feedback to users (athletes, patients) and/or experts (instructors, coaches, therapists) and implement all the elements and functionalities from Figure 1. Research papers that study only a single part of a BMF system or do not implement a complete feedback loop are excluded from this review, regardless of the plans of the authors to finalize their feedback system in the future. Various technologies can be used in real-time BMF systems, as this is a field of ongoing research with fast and changing development, only future studies will show which of the technologies are the most suitable in certain sport or rehabilitation activities.

We have systematically studied the relevant published articles in the field of real-time BMF in sports and physical rehabilitation over the last 20 years. This has given us a good insight and understanding of the field of real-time BMF, its evolution over time, and state-of-the-art solutions. 

The main contributions of this review are: (a) a systematic analysis of the field of real-time BMF systems in sports and physical rehabilitation, (b) an overview of the field based on different building blocks of BMF systems and their technological basis, (c) a critical review of the solutions and studies that we have identified as promising and best practices, presenting novel solutions and remarkable implementations, (d) highlight certain limitations and problems of studies in this field, and (e) an assessment of the state of the real-time BMF field.

## 2. Methods

To thoroughly investigate the field of real-time BMF systems, we conducted a systematic literature search and review. We followed the PRISMA framework for reviews and meta-analysis, focusing on those parts that were relevant to the technically oriented aspects of our study [16]. We searched three well-known databases: Scopus, PubMed, and Web of Science (WoS). The last search was performed on 3 December 2021. We considered both seminal studies and recently published state-of-the-art research. We were interested in real-time BMF systems in sports and physical rehabilitation involving concurrent, cyclic, and event-driven feedback, as defined in the Introduction section. Additionally required inclusion criterion was the implementation of a closed-loop BMF system. BMF systems involving only terminal feedback and post-processing were not included in the review. The literature search was conducted using a search term designed to capture and include as many papers as possible that fit our study:

(Sensor* OR System* OR Application*) AND (Kinematic OR Inertial OR Motion OR Force OR Pressure OR Accelerometer OR Gyroscope OR Strain*) AND (Feedback OR Biofeedback) AND (realtime OR “real time” OR real-time OR concurrent OR Instant) AND (Sport* OR Rehabilitation).

The same search string was used in all included databases, using the built-in advance search and generally searching by title, abstract, and keywords. We searched for articles throughout the life of the databases. 

**Figure 2:**
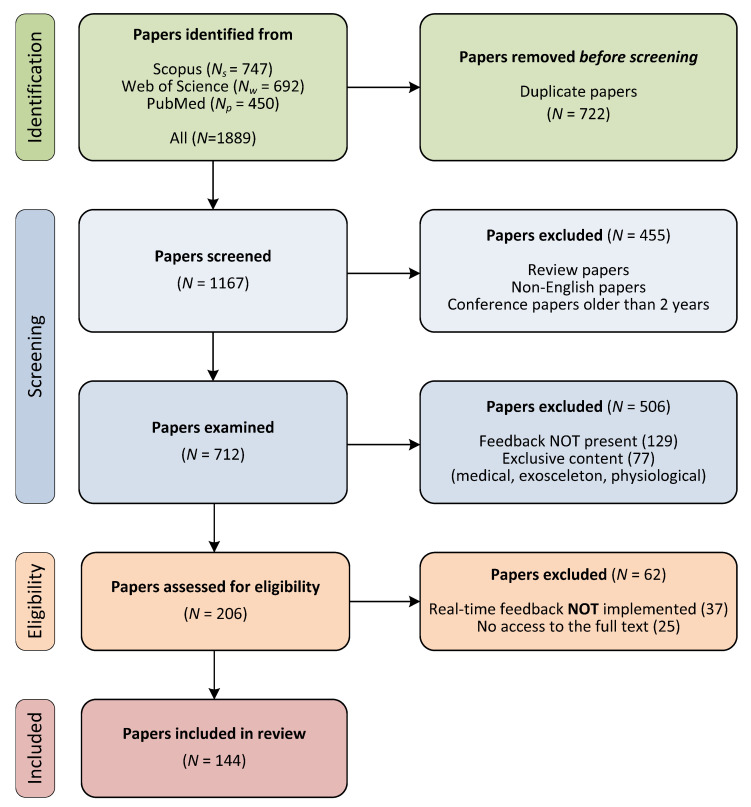
Results of database search and the selection of papers. A total of 1889 papers were found in the three databases searched, 1167 unique papers were screened and 144 of them were finally included in the review.

The database search and paper selection process are shown in Figure 2. We found 1889 papers from all three databases: 747 from Scopus, 692 from WoS, and 450 from PubMed. All papers were combined and 722 duplicates were identified and removed, giving us 1167 unique papers as input to the screening process; for the deduplication we used Zotero software and manual inspection of the papers.

In the first step of the screening process, which was based on bibliographic parameters, research papers and conference proceedings from recent years were included. Review papers, non-English language papers, and conference papers older than 2 years were removed, leaving 712 papers for the second step of the screening process. During the examination step, we used the title, abstract, and keywords to make a rough assessment of the eligibility of papers. We narrowed the number of papers by including only those that stated in the abstract or title that they were providing feedback to the user. We excluded all papers that did not state that a feedback loop was implemented and all papers that fell outside the scope of BMF in sport and physical rehabilitation. We excluded papers that were found by our search string but fell into the category of medicine, where the term “(bio)feedback” is often used in a different context, as a remedy or cure for various diseases. We also excluded works in which the rehabilitation process is performed with external assistance from various harnesses, e.g., exoskeletons, because as BMF is defined in [2,7], it is the person’s own cognitive abilities that cause the changes in movement, not the equipment. Since the focus of this work is on biomechanical parameters, we also excluded papers in which users received feedback about their physiological parameters. In total, 506 papers were excluded: 129 that did not implement a feedback loop and 377 papers that did not fall within the BMF scope studied, e.g., papers that included a BMF loop but were from the fields of medicine, exoskeletons, or others.

The remaining 206 papers were reviewed in detail for eligibility as part of our evaluation process. At this point, we examined the full texts of the manuscripts. The main inclusion criterion for the review was the appropriate implementation of a real-time BMF. We identified and removed 37 papers that had not implemented a closed-loop real-time BMF and thus did not meet one of the main inclusion criteria. We did not have access to additional 25 full-text articles. This left the final 144 papers that we included in our review.

**Figure 3:**
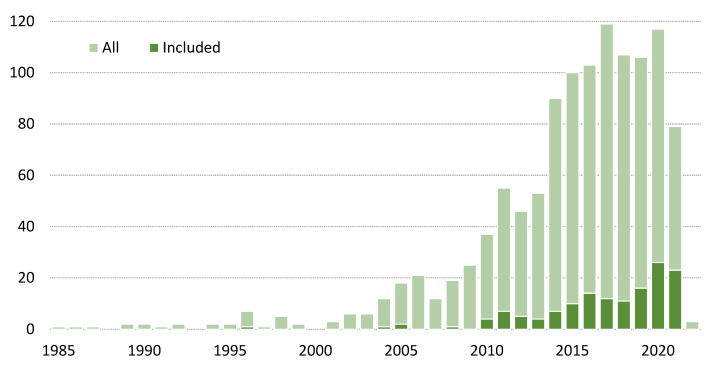
The frequency of articles by year of publication. All unique papers found (*N* = 1167) are shown in light green, papers included in the review are shown in dark green (*N* = 144).

The graph in Figure 3 shows the frequency of articles by year of publication. The first screened publication dates back to 1979 (not shown in the graph), and as can be observed, the intensity of research in the field of real-time BMF systems and applications in sports and physical rehabilitation began to increase rapidly around the year 2000. According to the results presented in the Results section, the most relevant research in the studied area has been conducted since 2010.

**Figure 4:**
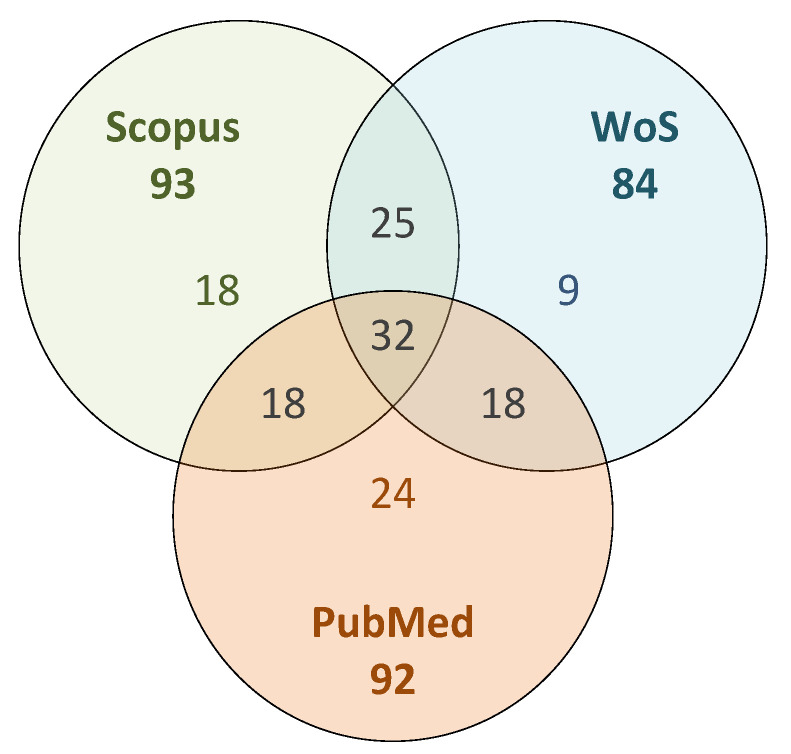
The distribution of included papers among the search databases. The values in bold show the number of included papers found in each database. The values in the cross sections show the number of included papers two or all three databases.

The distribution of included papers among the search databases is presented in Figure 4. For the purposes of this discussion, it is important to note that Figure 4 shows the total number of included papers for each database before the deduplication process. These numbers are in bold, next to the database name, and are the sum of all other numbers within the same circle. It is interesting to note that the papers that met all of the inclusion criteria and are therefore considered as more relevant, are relatively evenly distributed across all three search databases. Looking more closely at Figure 4, we can see that 32 papers are included in all three databases, 61 papers are included in two of the three databases and 51 papers are represented in only one database.

All included papers were carefully and thoroughly examined based on the set of research questions, designed to provide answers to concepts, processes, elements, parameters, and other important facts about the BMF systems studied:

- What type of activity (sport or therapy) is being performed?
- What type of sensor or sensor system is used to acquire movement parameters?
- How many sensors are used and where are they placed?
- Is sensor fusion being used?
- What type of system architecture is used in the implementation?
- Where and how is data processing performed?
- What type of modality is used for feedback?
- What type and technology is used for communication?
- What is the intended use of the system (aim, user, environment)?

During the review of the papers and based on the research questions presented, a table was created with relevant information about the papers examined and the features of the BMF systems and applications. Some of the main features are: field of study, activity, sensor type, system architecture, processing devices, feedback modality and devices, communication technology, and usage environment. The feature list is exhaustive, and it should be noted that many papers did not provide enough information to fill in the data for all the features. We acknowledge that not all aspects of the BMF and user studies were examined. It is clear from the research questions and studies that this review focuses on the technical aspects of BMF systems and applications. Therefore, we also acknowledge that the studies reviewed may contain author bias regarding BMF success and benefits. We leave the assessment of the success and benefits of BMF to experts in the fields of sports and rehabilitation. We believe that our screening and examination process, which was conducted collaboratively by the authors and curated by the first author, was transparent and the inclusion criteria (real-time closed-loop BMF) were clear and rigorous, so no major biases can be attributed to this work.

## 3. Results

The papers included in the review examine a number of aspects of BMF systems. They examine different features and offer different solutions to different problems of BMF. We were careful to evaluate only those studies that met the inclusion criteria by clearly demonstrating the implementation of a real-time BMF system.

One of our goals was also to learn more about the technological aspects and the current state of the art in the field of real-time BMF systems and applications in sports and physical rehabilitation. We were particularly interested in sensing, feedback, and processing technologies that enable real-time operation of BMF systems. 

After a thorough review of the included papers, based on the research questions and a set of BMF system features presented in the Methods section, we developed criteria and categories that allowed us to group similar studies, identify differences between them, and compare them in different ways.

### 3.1. Activity

**Figure 5:**
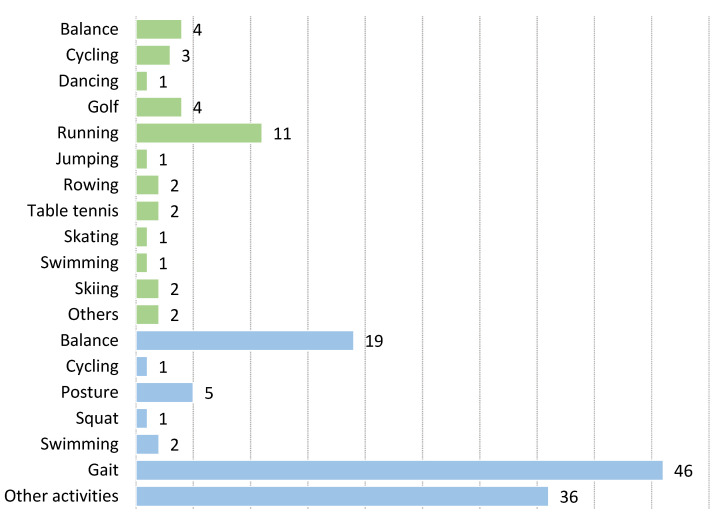
The number of papers according to different activities. Bars in green represent sport activities, bars in blue represent physical rehabilitation activities.

We divided all activities into two main groups: sports and rehabilitation, as listed in Table 1 and shown in Figure 5. These two main groups were further subdivided according to the specific activity presented in the study.

Compared to sports studies, there are more than three-times as many papers in the field of rehabilitation. However, we managed to divide the 33 sports studies into 12 subgroups and the 111 rehabilitation papers into 7 subgroups. The subgroups with the most papers are *gait* (*N* = 45) and running (*N* = 11) in the rehabilitation and sports groups respectively. Papers dealing with various aspects of walking (gait) are the most represented group. The *other activities* group includes papers dealing with specific physiotherapy exercises required for patient recovery, with the study of specific physical rehabilitation exercises using BMF as the link between them. The prevalence of gait and physical therapy exercises is striking.

### 3.2. Sensors

**Figure 6:**
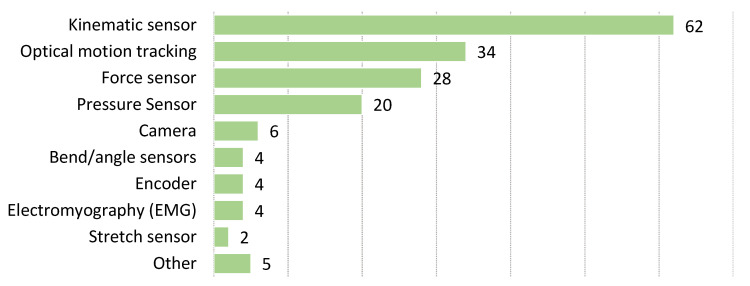
The number of studies using different sensors. Because some studies use more than one type of sensor, the total number is greater than the number of papers included in the review.

Researchers use various sensors to measure and evaluate human movement. We consider technologies ranging from a simple wearable device to a complex professional system for precise kinematic tracking. We evaluated and classified sensors based on their type, sampling frequency and body placement. The sensors were classified into eight groups, which are listed in Table 2. The distribution of contributions among the different sensor groups is shown in Figure 6. Kinematic sensors are the most represented with 62 contributions. In fact, most studies use the term Inertial Measurement Unit (IMU). IMU includes accelerometer, gyroscope, or both. In many studies, a magnetometer is added to the IMU sensor combination. It should be noted that the magnetometer is not a kinematic sensor, but it is essential for providing information for accurate localization in space. Force sensors are used in 28 papers; they are usually used as force plates or in some other load cell application. Pressure sensors, on the other hand, are less precise but can be worn on the body, normally in the form of force-sensing resistor. They are used in 20 studies. 

Optical motion tracking is widely used in kinematic studies as the primary or validation system. One type of commercial optical motion tracking system is used in 34 papers. These include professional systems with markers as well as commercially available devices such as Kinect, which uses stereoscopic infrared vision for motion detection and tracking. Some studies (six) use video cameras, usually combined with another sensor.

Other notable sensor types include bend or angle sensors, used in four studies, stretch sensors used in two studies, and angle encoders used in four studies. Although studies using electromyography would normally be excluded from our review, three papers use it in conjunction with other sensors. One study uses it exclusively, but we consider this work important because it addresses other aspects of BMF. It is quite common for studies to use more than one type of sensor, but sometimes it is difficult to distinguish which sensors or which combination of them provides the input for the feedback. Table 2 lists all the sensors mentioned in the papers studied. 

**Figure 7:**
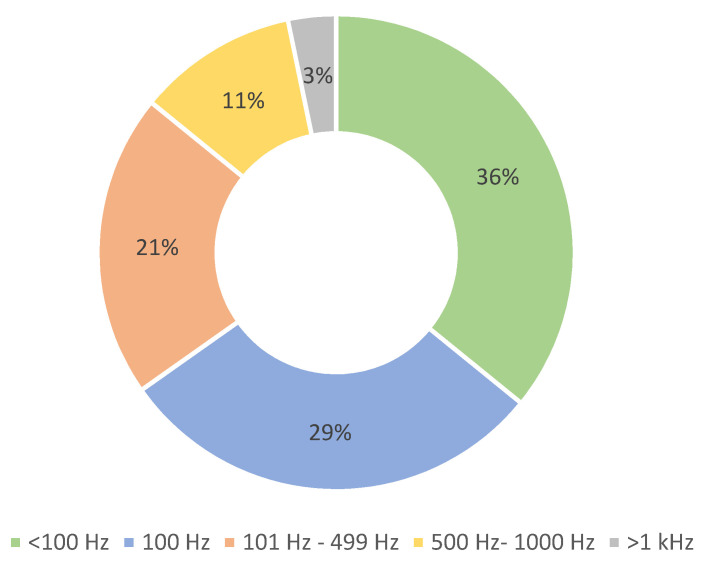
The number and percentage of studies in which sampling frequency was used in specific frequency ranges. This information was not reported in 37% of all papers reviewed.

A very important aspect of the technical implementation and operation of a BMF system is the **sampling frequency**. We do not distinguish between the sampling frequency of the system and the sampling frequency of its sensors, since this information is usually not clear in the texts of the papers; 54 papers lack information on the operating frequency. The distribution of sampling frequencies is listed in Table 3 and shown in Figure 7. We divided the studies into four representative groups to obtain a general overview of the field. There are 32 papers with sampling frequencies ranging from 1 to 99 Hz. The most commonly used frequencies are between 100 Hz and 499 Hz. In total, 46 papers use a sampling frequency within this range, 27 of which use exactly 100 Hz. Sampling frequencies of more than 500 Hz are used in only 13 studies. Only one case [55] described more than one sampling frequency. Table 4 lists the papers according to the groups presented.

**Figure 8:**
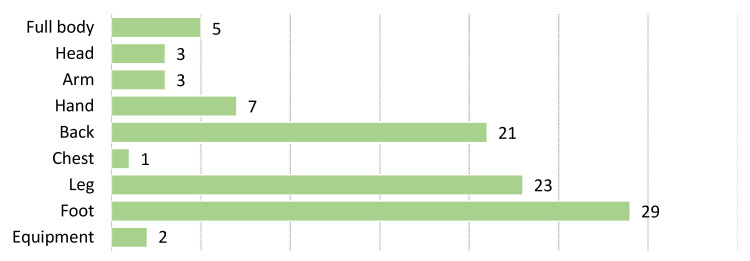
Wearable sensor placement on the user’s body or equipment.

In biomechanics, we are interested in the movement of the body and its parts. This makes the **position** of the sensor one of the most important elements in measuring motion. Other authors have already studied the aspects of different sensor location placement [161,162]. Our results agree with their findings and surveys. If you are only measuring general activity, the position of the sensor body is less important. The location of sensors is listed in Table 4 and shown in Figure 8. The most representative body areas are legs and feet, which fits well with the most represented activities from Table 1. In two cases, the sensors were placed in or on the sports equipment. In 59 cases, we were unable to determine the exact location of the sensor because no information was provided in the study or they used a motion measurement system that did not require positioning of the sensors on the user. This was primarily because the sensor used was not a wearable sensor, i.e., optical tracking, camera, or force plate. 

Multiple sensors were used in the studies. This is especially true for studies using the sensor fusion concept. This is the ability to combine information from multiple sensors to measure and calculate motion. **Sensor fusion** is used in 21 papers [17,25,29,47,51,53,54,61,62,63,64,76,81,90,110,115,119,139,149,157,159].

### 3.3. System Architecture and Data Processing

BMF systems and applications all follow the basic idea presented in Figure 1, but their implementations in terms of system architecture may involve significant differences and some limitations. We distinguish between two basic system architectures: compact and distributed. In the compact architecture, sensors, processing unit, and actuators are housed in the same device, or in only a few devices that are close to each other and seamlessly interconnected, often by cables. Such systems are worn by or located near the user. In distributed architecture, the sensors, processing unit, and actuators are in different locations. In most cases, the sensors and actuators are worn by or located near the users, and the processing unit is located in a remote location.

**Figure 9:**
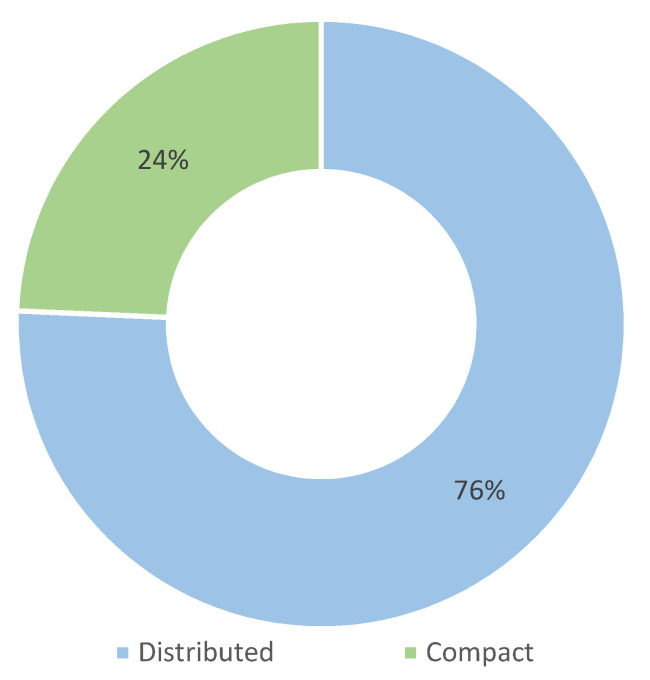
BMF system architecture.

Our review found that most of the included studies implement a distributed version of the BMF system architecture. From the list in Table 5 and the chart in Figure 9, we see that less than a quarter of the implemented systems are classified as compact.

**Figure 10:**
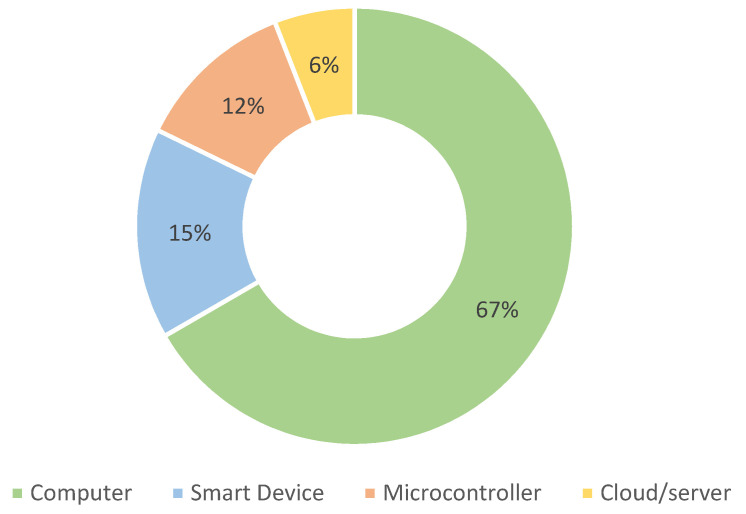
BMF system or application processing device type.

Sensor data must be processed and transformed appropriately and in a timely manner before feedback information can be presented to the user. As listed in Table 6 and shown in Figure 10, in most cases the processing is carried out using a computer system, e.g., a PC, laptop, or similar. The second most represented group is smart devices (smartphones, tablets, smart goggles), followed by embedded systems, which are purpose-built devices with integrated microcontrollers, and cloud/servers.

Regarding the main processing method used, digital signal processing (DSP) is mostly used for signal analysis and feedback information conversion, e.g., visual feedback generation. In only four papers [22,42,151,154], machine learning algorithms are described and used for data processing. Moreover, in some examples, processing is performed on multiple devices [24,72,97,112,157,158,159].

### 3.4. Feedback Modality and Actuators

**Figure 11:**
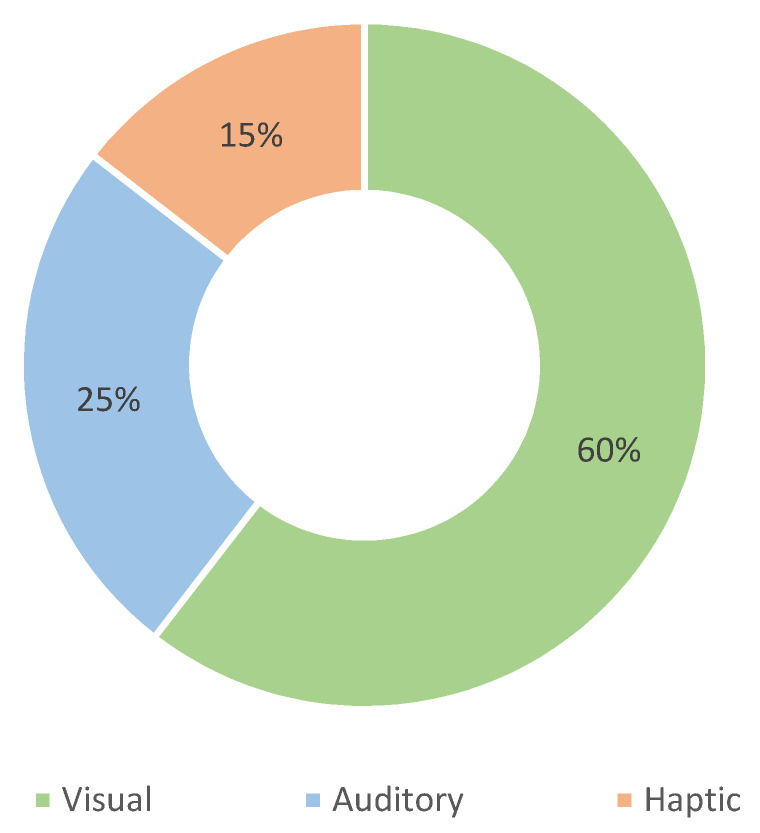
Utilization of feedback modalities in studies from all included papers.

**Figure 12:**
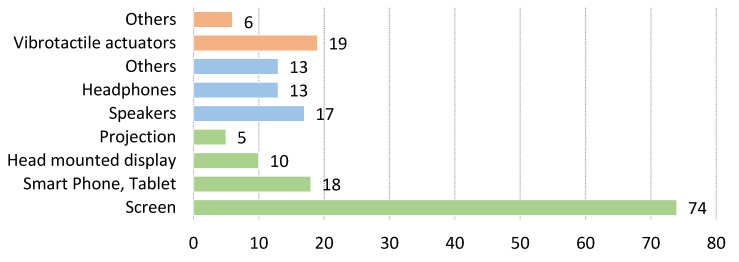
Feedback device usage according to different feedback modalities. Visual feedback devices are represented in green, auditory in blue and haptic in orange.

The necessary functionality of any BMF system is its ability to provide useful feedback information to the user; in our case, the information about a user’s movement or performance. Feedback information can be presented to the user with a variety of actuators and using available modalities, as described in the Introduction section. The feedback modality is influenced by the medium and the human sense it stimulates. This means that visual, auditory, and haptic feedback interfaces use vision, hearing, and touch as modalities, respectively. The use of these modalities is presented in Figure 11, with nearly two-thirds of the studies using visual feedback. The distribution of feedback devices used is shown in Figure 12.

Table 7 lists the different modalities and types of devices used for feedback. We found 104 studies describing the use of visual feedback interfaces, which can be divided into four groups: screens, smart devices, projections, and head-mounted displays. The latter consist of virtual and augmented reality headsets and isolated use of various types of wearable displays. Visual feedback interfaces vary in size and offer different content. Smart devices can be further divided into smartphones and tablets.

In 43 studies, interfaces with auditory feedback are used. Devices that provide auditory feedback are divided into three groups: headphones, and others that could not be specifically categorized or use other technologies. In some papers, the source of auditory feedback is not described.

The haptic modality is the least used and is described in only 25 of the papers studied. Feedback is provided via vibrotactile actuators attached to a specific part of the user’s body. Six works use a technology or stimuli other than vibration to provide a haptic interface.

Some studies use more than one modality or test different modalities. In two papers, all three modalities are used [83,101]. Visual in conjunction with auditory is used in 20 studies [26,32,38,39,40,47,81,84,89,98,114,116,121,122,126,127,149,153,154,160], while visual is used with haptics in only three papers [41,65,109]. The combination of haptic and auditory feedback is studied only once [100].

Another important aspect of providing feedback is to whom it is delivered. That is, how is the feedback information conveyed to the user. In 123 studies, feedback information is delivered directly to the user, i.e., the patient or athlete. If the user receives the information indirectly, e.g., through a coach or therapist who receives the information in real time, we consider this as an indirect path, which was the case in seven studies [45,46,49,72,77,124,151]. Some papers [22,24,30,69,76,103,125,132,134,136,141,160] offer both options.

### 3.5. Communication

Sensors, processing units, and actuators must communicate with each other. They can use a variety of different communication modes and technologies. In Table 8, we first distinguish between wired and wireless communication. Wired communication was used in 31 papers and wireless communications in 68 papers. Specific sensor systems are used in 47 studies, so this information is irrelevant or not shared. Wireless communication is further divided into specific technologies. A special case was the use of LoRa in [45], but, in general, most research papers use technologies operating in the 2.4 GHz industrial, scientific and medical (ISM) band. This is true for Wi-Fi and Bluetooth, as well as for most custom solutions, which are categorized as “Other”. Some papers do not describe the exact operation of their wireless solution or use commercial systems that do not disclose this information. 

### 3.6. Primary User and Environment

Another important aspect of the BMF system is its primary user. This can be either an end user (patient or athlete) or an expert working with the user. Patients and athletes are considered primary users of the BMF, if they can use it independently, without the presence of an expert. If the operation of the BMF system requires an expert, we consider the expert as the primary user. In such systems, the end user receives direct feedback, and the expert monitors and manages the system and the training or therapy procedures. 

**Figure 13:**
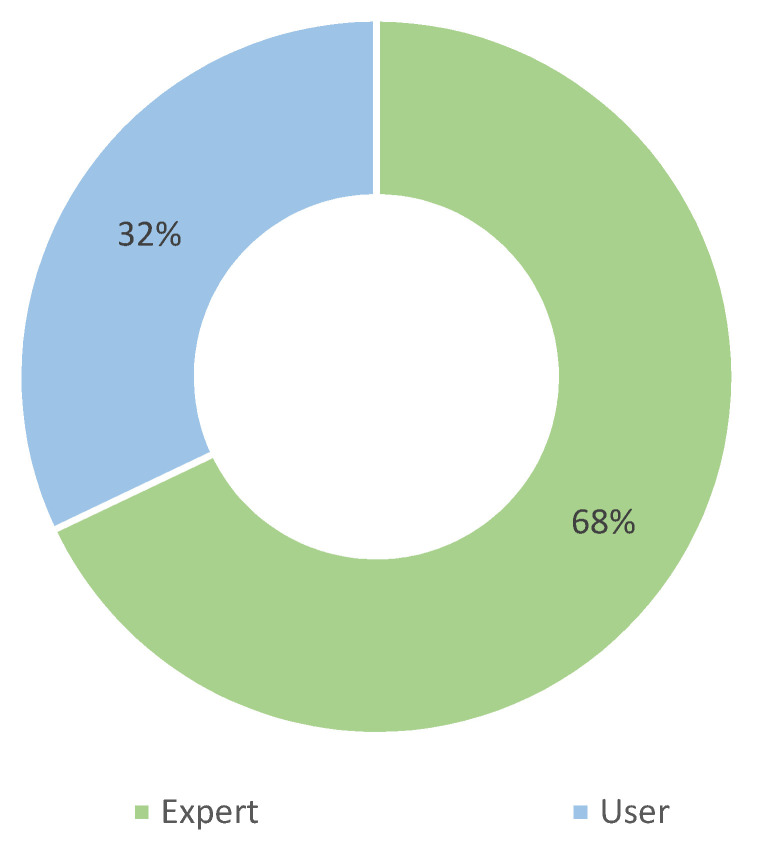
BMF applications prevalence according to the primary user.

The choice of the primary user establishes the difference in terms of the purpose of the application and the complexity of the system. Table 9 and Figure 13 show that studies with applications designed for experts are more than twice as common as those aimed at end users. In 12 cases, we consider both experts and end users as primary users because the application can be used in both scenarios, by the user alone or under the supervision of the expert.

**Figure 14:**
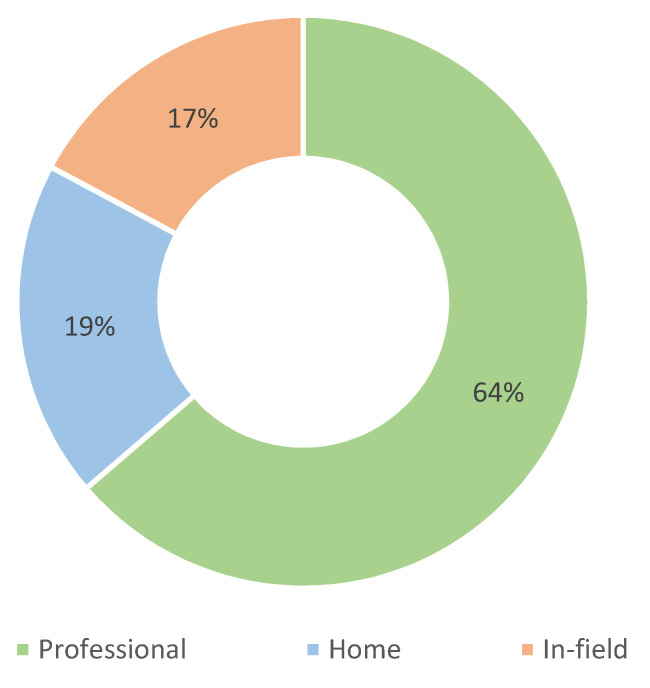
Intended BMF application use environment.

The application environment is another issue related to BMF systems. Some of them have limitations or specific operating parameters that tie them to a particular professional environment, while others can be used in a variety of environments. We identified three different environments, listed in Table 10 and shown in Figure 14. Of all included papers, 100 are tied to either the professional or laboratory environment, meaning that once a system is in use, it cannot be moved or can only be moved with difficulty. A total of 30 applications can be easily operated by the user and used at home, and 27 applications are intended for field use, i.e., they are portable but cannot be easily used by their users only. A combination of different application environments can be found in 13 papers [25,33,53,92,97,100,101,133,134,135,137,141,142].

## 4. Findings

Our research in the field of real-time BMF systems, searching three well-known databases Scopus, Web of Science, and PubMed, yielded a large number of articles. Out of 1167 unique entries from all three databases we started with, we reviewed and carefully examined 144 papers. In our opinion, 24 of these papers show novel solutions and remarkable implementations of real-time BMF systems [21,24,25,27,31,33,35,36,37,47,51,54,76,83,88,100,108,113,118,119,124,137,156,159] and they also show clarity in reporting system design and results; they are presented in more detail in this section. Another 34 papers show the significance of some part of the BMF system: sensor, actuator, design or implementation [17,18,20,22,23,28,32,41,43,44,45,49,58,68,71,72,77,81,82,86,93,97,103,104,105,114,117,120,123,131,133,149,152,153].

### 4.1. Activity

The majority of the papers included in the review are part of physical rehabilitation activities and only a minority of the papers are from the field of sports. This is not surprising, as the complexity of movements and the needs of the users in these two areas differ significantly. At the current state of development, BMF may provide greater benefits to a patient with physical impairments in terms of rehabilitation exercises and in a laboratory/hospital setting while performing a specific, usually repetitive, movement, than to an athlete using a series of complex movements for training and execution of a specific sport. The prevalence of activities involving less complex (repetitive) movements is confirmed by the fact that the repetitive movement of walking or running is studied in 56 of 144 (39%) papers, 10 of which examine running as an athletic activity. Moreover, physiotherapeutic exercises in 36 papers (25%) consist exclusively of repetitive movements that require careful execution. We found only a few papers with activities that are much more concerned with complex movement studies. Chun et al. [25] and Koyama at al. [27] used different approaches to provide feedback while learning golf. Both studies created a golf coaching system. Study [25] uses multiple sensors on a golf club and user, combining this data with an optical tracking system to create visual and auditory feedback. The main purpose of this application is to help users recognize their faults and improve their skill in golf motion. Paper [27], on the other hand, shows a universal wearable sensor and feedback system, but demonstrates the usability of that system in both running and golf swing with auditory feedback. They use a special kind of wearable optical fiber sensor worn on the body to track motion. They are able to provide real-time auditory feedback to trainers in their motion. Kuwahara et al. [47] developed a snowboard training system, with the main purpose of training novice boarders with visual and auditory feedback. Using multiple sensors to measure the center of pressure and provide feedback about the actual and ideal center of pressure. Their main contribution shows the difference between using auditory and visual feedback and using no feedback. Grosshauser et al. [24] developed a wearable system for sonification in dance learning and training, with the use of auditory feedback that provides information to the user and trainer that is otherwise not available. They can use this information to improve and correct a dance move. They show that sonification helps both dance students and teachers to reduce strain on the body and improve movement. Gandolla et al. [76] studied swimming and rehabilitation in water. They designed a system capable of linking water momentum to BMF using a multipoint network of kinematic sensors to personalize and objectify water exercises. They use a body wearable sensor suit with kinematic sensors; this movement is shown to the user via visual display. With this system, they aim to increase the user’s awareness of their body position and movement, and allow the therapist to monitor the user.

### 4.2. Sensors

The evaluation of each movement begins with a motion sensor that provides the input for the BMF systems. Primarily, BMF systems and applications use some type of kinematic sensor with accelerometer, gyroscope, and/or magnetometer. However, other sensors such as optical motion capture, force and pressure sensors are also used. Optical motion capture is used by Sanford et al. [156] to detect the motion of a patient during an assistive feedback exercise. They also use force sensors and electromyography to describe and understand the motion, and try to improve muscle activity using concurrent visual feedback. They show that visual feedback in conjunction with precise and complex optical motion capture can be beneficial in rehabilitation exercises. Force sensors are also used in other remarkable studies [31,33,88,124]. Donatell et al. [124] used a force sensor attached to a dedicated spine measurement device to measure flexion in conjunction with an optical sensor, trying to create a therapeutic tool to monitor and enhance medical exercises of patients with chronic lower back pain. Four strain gauges were used to measure the flexion of the spine as well as an optical sensor for the measurement of side-to-side movement. This system measures deflections of the spine form a natural position with inexpensive technologies, while being comfortable and providing feedback to the user. Others used force sensors in an insole. Dingwell et al. [82] describe real-time feedback using a treadmill with two integrated force plates to provide real-time visual feedback to the user. The main idea here is to simplify and objectify measurement of gait, which would take more than an hour using conventional (manual) methods, to less than half a minute. This was the first solution we found that attempts to provide real-time feedback to the user. A number of recent studies use similar solutions for motion analysis. Other studies [33,83,100,113,118] also use pressure sensors to measure foot loading during running and gait studies. In [24], a special sensor equipment is used to measure dance patterns with multiple sensors: IMU, goniometers and pressure sensors. The application provides auditory feedback to both the dancer and the trainer. Pressure sensors are attached to the insoles, goniometer to the knee and IMU along with electronics to the foot. The authors point out the advantages of auditory feedback, and these probably outweigh identified shortcomings in the attachment of the sensors. Kuwahara et al. [47] developed a custom snowboard with pressure sensors and a IMU sensor under the snowshoe to measure the user’s posture. They used this custom-built sensor equipped snowboard to confirm the feasibility of enhancing motor learning with visual and auditory feedback. The system was able to successfully assist the athlete in correct weight transfer, minimizing the difference between perceived and actual movement. In [27], a customized sensor solution with optical fibers was presented to provide whole-body motion analysis. They used hetero-core fiber optics attached to the stretch clothing. They show that changes in light leakage from bent optical fiber can be used to track motion. The system shows the early stages of future sportswear with integrated wearable fiber optic sensors. Some research groups [20,43,68,71,133] have successfully used commercial home entertainment sensor technology for their study. They use either stereoscopic cameras, commonly used in home entertainment, as optical motion tracking devices, or commercial force plates as sensing devices. 

We can conclude that the sampling frequencies in all papers were appropriate for real-time operation in their respective domains. Since we only study BMF systems that implement a complete feedback loop, we emphasize other parts of the system that are often overlooked by other authors. The term “sensor fusion” should be commented on because different authors use the same term in different contexts and some do not use this terminology despite its common adoption. While some authors often use a specific combined use of an accelerometer and a gyroscope to provide absolute orientation angles such as Euler angles or quaternions, here, we define sensor fusion as the use of two or more synchronized sensors and algorithms to achieve a result that cannot be achieved with a single sensor. For example, the result of an accelerometer and a gyroscope combined with a Kalman filter can be described as sensor fusion. However, using accelerometers and gyroscopes to separately evaluate motion would not be considered sensor fusion, even if multiple synchronized sensors were used. We have been careful to highlight only papers that use sensor fusion in their text in the proper context [51,54,76] or describe algorithms that we can consider sensor fusion [25,47,119,159].

### 4.3. System Architecture and Data Processing

BMF systems and applications have different architectures. We distinguish between distributed, that predominates, and compact architectures. There are several use cases in which each architecture paradigm offers specific advantages. In general, they differ in the complexity of the system. In most cases, compact BMF systems consist of a single device, which also performs the processing, with one or more sensors and/or actuators connected by wires or wirelessly. Compact systems are portable. 

There are four noteworthy examples of compact systems [33,37,100,159] that function as a single unit, with sensors and actuators connected directly to a processing device via cables, but do not restrict the user’s movement. Cheung and Davis [33] and Khoo et al. [100] use a wearable device connected to sensors and an actuator that provides audio feedback. The authors of [33] use a simple insole sensor with a feedback device connected to a buzzer. When the hill strike occurs, the buzzing sound is generated. Runners were instructed to minimize or eliminate the buzzing. On a small sample of runners, they were able to prove that this system improves landing patterns and reduces pain in the foot. The authors of [100] work with stroke patients. They use an insole with multiple force sensors. They show and prove that by using auditory feedback, the shift in the body weight form hill to front foot is faster than without any feedback. Van den Berghe et al. [37] use a smart tablet in a backpack as a processing device with wired sensors and connected headphones for auditory feedback. Feedback is generated based on the running rhythm and by adding noise to the background music. Depending on the running steps per minute, a song with similar beats per minute is played. As the running rhythm changes, so does the rhythm of the song. Pink noise is added to the music based on the values of average peak tibial acceleration. Athletes with BMF were able to significantly reduce peak tibial acceleration compared to athletes without feedback. Similarly, Ashapkina et al. [159] developed a custom haptic device with integrated kinematic sensors that can be operated as a stand-alone system or connected to other devices. The main purpose of this device is to provide real-time correction of the specific physical exercise. They use multiple vibrotactile actuators attached to a belt to provide a variety of possible feedback options depending on the exercise and algorithm used. The compact paradigm is also found in [35,83,88], but with wirelessly connected sensors and actuators. Compact systems typically have processing devices that are either smart devices (phones or tablets) or microcontrollers. 

Distributed systems, on the other hand, use multiple sensors and actuators, with processing usually carried out on more powerful computer systems, such as personal computers, laptops, and the cloud. Distributed systems used in sports should use wireless communication between the processing device, sensors and actuators. In rehabilitation, wired connections are viable for relatively static activities. Remarkable systems using wired solutions include [124,156], both with processing on a dedicated computer. Two studies [31,108] use a smartphone or tablet as the main processing device, with the sensor devices connected using Personal Area Network. Four notable distributed architecture studies [24,27,47,118] used low-power devices with microcontrollers for main signal processing, although some also used computers in the setup. All other remarkable distributed systems used computers as the main processing devices. Several processing algorithms are used in the studies. Some common algorithms that we were able to identify from the texts are: filtering [25,27,35,47,51,54,76,108,118,137], projections [25,118], modulation [24,37] and thresholding [36,83,88,100,108,124]. Some combinations of those algorithms and other more advance algorithms are also used [25,119,159].

### 4.4. Feedback Modality and Actuators

From the results, it appears that **visual modality** is most frequently used by researchers. This is not surprising, since humans can understand visual information with minimal learning. Some of the most advanced visual feedback systems published in the last two years use screens or displays [21,76,119,156]. Yokota et al. [21] present a system for indoor cycling training. With multiple sensors attached to the user’s leg and visual feedback that visualizes the sensor signals in time as a virtual circular map and compares the target movement with the user’s actual movement. This visualization allows users to easily imitate the desired movement by displaying the difference between movement trajectories in front of them. This allows users to adjust their motion to the motion of the expert in real time. A simple visual representation of human motion is presented by Gandolla et al. [76] when performing aquatic exercises. When performing rehabilitation exercises in water, users can follow the movement in real time on a display in front of them. Liu et al. [119] have developed a visual feedback display for pediatric patients with cerebral palsy. They use a type of scoreboard with a visual interface with target patterns for patients walking on a treadmill. This type of feedback is intuitive to understand and can be used in the recovery of children. This system can enhance pediatric gait training, but the system has only been tested by a single child with cerebral palsy. Similarly, Sanford et al. [156] used a large visual display to provide feedback to the patient during various static rehabilitation exercises indicating the difference between the target and the user’s movement. They tested the differences between styles of visual representation from simple to complex and show that concurrent visual feedback is beneficial during squat exercises and that the most complex representation of the body on the screen shows the greatest success with users. In [104,110], a special type of immersive semicircular display is used to create a virtual environment with visual feedback while performing a treadmill walking study. 

Early studies [93,137], provide some insight into how researchers envision the use of augmented visual feedback. The main idea is a head-mounted display with information about the user’s spinal geometry using kinematic sensors. None of these head-mounted displays would be considered augmented reality by today’s standards, as they simply have a semi-opaque small display in front of the user’s eyes. However, two papers [93,137] show that the wearable paradigm was being explored even before the technology was ready for it. Recent examples of advanced augmented visual feedback solutions can be found in [47,84,110,138], with various head-mounted devices. In [84,110], Microsoft HoloLens is used as augmented reality glasses that overlay virtual objects and visual information into their actual surroundings. On the other hand, [138] provides a different experience using HTC Vive virtual reality for rehabilitation. Promising steps are being taken by Kuwahara et al. [47], providing real-time visual feedback to the snowboarder using both head-mounted goggles and auditory feedback via headphones. We see this as a continuation and another opportunity to investigate smart sports devices with BMF, which our research group proposed a few years earlier [49]. A simple but more wearable solution for visual display is the ability to use a smartphone or tablet as a means for feedback. Mobile BMF applications can be found in [18,22,72,77,83,153]. They also allow the use of other modalities as they already have actuators for auditory [153] and haptic feedback [83]. 

**Auditory** information can be stimulated by different sources, but in our study, we found that only loudspeakers and headphones were used, depending on the activity, the setup, and the information offered. In two studies [33,100], speakers in the form of piezoelectric transducers were used to provide audio information with buzzing sounds. In both studies, insole sensors and simple processing devices were used to provide **auditory feedback** to the user about their walking and running performance. They can be considered initial studies because no sound shaping or audio processing is required. The use of speakers or headphones makes a big difference when considering the usability of a system. If the BMF systems is used in a professional setting, speakers [24,27,32,81] can be used because both the user and the expert can hear the audio feedback. In a more personal or portable system, headphones [28,37,44,47,105,108] are usually used. On the other hand, in four studies [31,83,113,153], the smartphone is used in conjunction with a dedicated app as an auditory feedback source and processing device. The experiments in [24,44,105,113] on sonication, which provided a relative change in tones as a function of the simultaneous responses of various sensors, are of great interest for further development. 

Finally, information can be provided to the user through the sense of touch as **haptic feedback**. There are several ways to perceive information via the sense of touch, and technologically there are several options as well. In our research, we found that almost all researchers use vibrotactile actuators in the form of eccentric vibration motors. However, there are differences in the way these actuators are arranged on a body. Ashapkina et al. [159] present a wearable feedback device with multiple vibrotactile actuators arranged around the user’s wrist, allowing multiple options for providing feedback information with different patterns and intensities. Other authors provide haptic feedback with multiple actuators attached to the user’s body. In the walking application [35], two vibromotors are attached to each leg, and in [51,54] they are attached to the left and right sides of the user’s hip for posture control. A single actuator or actuators in a single location are used in [36,88]. In both cases, the actuators are attached to the wrist in the form of a haptic bracelet, in the first case as feedback during walking and in the second case for stroke rehabilitation. A different type of haptic actuator is used in [100]. The authors use electro-tactile feedback given in the form of an electrical stimulus that produces a tingling sensation on the user’s skin.

### 4.5. Primary User and Environment

Feedback can reach the user in two basic ways, **directly** or **indirectly**. Depending on how the BMF system or application is implemented, feedback information can be received in real time by the user or by the expert. Both use the BMF system concurrently, but the difference is who receives the information about the movement and how the user receives it. If the user receives visual, auditory, or haptic feedback directly, we consider this a direct feedback path. When an expert receives the feedback about the user’s movement and later relays that information to the user, either through a command or by changing system operation, it is an indirect feedback path. Sometimes both the user and the expert receive feedback at the same time. Interesting examples of indirect feedback can be found in studies of swimming [45,76,77], where a therapist or coach receives feedback and relays it to the patient or swimmer, while also attempting to provide direct feedback to the user. 

BMF systems vary in complexity and use case. The purpose of the BMF system depends on the sports or rehabilitation activity, desired outcomes, equipment, sensors, and actuators. We distinguish between three general environment or use cases: **professional**, **home**, and **in-field**. Some systems and applications can be used effectively in multiple use cases. The professional use case is defined as an activity that can only be performed with the help of an expert and in a controlled laboratory environment. This is usually the case for concept studies [21,25,36,100,104,119,156]. Systems intended for home use are often designed as wearable devices [31,33,83,97,100,137] or use commercial, readily available devices [20,68,71,133]. They are usually intended as self-sufficient systems that users can operate themselves, although an expert may need to be consulted for setup and initial instructions. Some systems are even designed for patient and therapist collaboration. The third option of in-field studies involves systems and applications that can work within the field range and do not require a predetermined setup. These generally include portable sensors with minimal setup [35,37,105,108,113,120,123,152], but also include studies that require a dedicated space such as a swimming pool or can operate outdoors [22,24,25,28,49,76]. Another important factor related to the use case is the primary user of the system. When the system is intended for experts to control and perform the study, the primary user of the system is an expert. On the other hand, if the system is intended for independent use by an athlete or a patient, we consider them to be the primary user. In general, both parameters—purpose and primary user—are related, but there are some exceptions: home and field applications are usually user-centered and can be considered a professional system only under special circumstances [25,33,97,108,133,137], but in some studies the primary user is clearly an expert, even if the system is used in the field [49,76,100,105]. On the other hand, systems with an expert as the primary user always operate in a professional laboratory environment, which is not surprising, since accurate and relevant data can only be obtained in this way. 

It is also worth noting that among the selected articles, there are several different research groups with multiple articles: Senanayake et al. [17,51,54], active in 2010 and 2011; Effenberg et al. [40,91,115,130,147], who have been active since 2018; Kuo et al. [70,73], who were active last year; Schwenk et al. [61,62,63,64], who were active from 2014 to 2016; Lee et al. [53,58,65], who were active from 2011 to 2015. Liu et al. [89,116], who were active in recent years; and Wang et al. [18,19], who were active in recent years.

## 5. Discussion

Our study shows that the field of BMF systems and applications is developing at a steady pace. Based on the work we have studied and our own experience, we present some technical guidelines, best practices, and technologies that we would recommend for future BMF application development.

The development of BMF systems focuses on physical activity, and everything related to the technical components is guided by the principle that human movement should not be obstructed by the equipment used. In that is the case, the BMF systems can bring benefits and be useful for the user, otherwise it can have opposite effects. Sensor, communication and actuator technologies must be able to work in the desired environment; equipment for swimming studies must be different from that used for gait rehabilitation or running. However, both walking studies and running applications can use similar or the same system for assessing two different movements [33,36,88,100].

In the featured articles, kinematic sensors are predominantly used, usually accelerometers and gyroscopes, and in some cases also magnetometers. This is not surprising, as these sensors allow us to capture different types of activities. They also have a small form factor, which makes them suitable as wearable sensors. When measuring running or walking, they can be used to capture the motion of the foot, leg, or center of mass. Some authors also want to record the load on the foot; in this case, they usually use pressure sensors in an insole. We have also observed a combination of both sensors [105]. The same set of sensors is often used for balance training. Advanced use of a kinematic sensor is demonstrated in the dancing application [24], where complex motions are captured and used as an input for auditory feedback. Wearable sensors in the form of smart clothing and textiles [161] will probably be very interesting for BMF studies in the future. In observed studies [27,153], there are examples of sensors that can be used to assess whole-body activity, and these will evolve in the future into smart textiles that use optical fibers and strain sensors for motion sensing. The general trend in this area is toward wearable sensors [9] and smart sport equipment [3]. The placement of wearable sensors is of paramount importance, as only the sensor placed in the correct location on the body or equipment is able to detect the desired motion, which is, however, application specific and cannot be generalized.

It is also worth noting that most papers use a sampling frequency of about 100 Hz. This frequency is suitable for capturing the majority of human motion patterns. Although, certain specific motions, such as impacts do require higher sampling frequencies, and with that more capable hardware [32,37,83]. The sampling frequency is also related to the communication bandwidth used. That is, if the devices cannot communicate faster and they are limited in regard to data transmission, the sampling frequency can be reduced to compensate for the limitations of the hardware.

If we want to achieve unobstructed motion analysis, the only appropriate way to communicate with the sensor is to use wireless technologies. From our experience, we can say that Bluetooth communication is less usable, although many authors use it, because of its bandwidth and range; nevertheless, due to easy commercial implementation with smartphones and computers, it is suitable for high volume commercial devices. Otherwise, any wireless technology can be used on different radio channels, but limitations due to water, obstacles, and environments that already largely occupy the radio spectrum must be considered when designing BMF solutions. For monitored activities in laboratory environments, wired solutions can also be used if the BMF equipment can be directly connected with cables without affecting the user. 

Sufficient processing power can be provided either by a remote device or by a wearable device. Basic digital signal processing techniques are currently used in the majority of studies, but researchers are already exploring machine learning techniques that can take advantage of potential information about movement by learning different behavioral and motion patterns of the user. 

There are different BMF system architectures depending on whether the application is intended as a tool for everyday use or if it is specifically used in a laboratory setting. If the BMF system is intended as an everyday wearable or training device, a compact solution in a single device is appropriate. However, if the system is intended for more precise and monitored rehabilitation or sports training, complex distributed systems may be used. As the BMF field evolves, more commercial end-user devices are expected to be available, and we expect them to implement a compact architecture. 

An interesting research question of BMF systems is the feedback modality, where further studies are needed on the effects of the different modalities in different situations. Once the movement has been captured and processed, relevant information about the motion is returned to the user via a specific modality. This can be achieved in a variety of ways. Researchers should decide at the beginning of BMF system development what information they want to convey to the user and how they want to accomplish this. The complexity and presentation of feedback information can vary significantly between modalities. Most of the papers in this review use visual feedback, which is not surprising as sight is a human’s primary sense. Visual information can be presented on a display and is, in most cases, self-explanatory to users, requiring minimal learning. On the other hand, auditory and haptic feedback require a certain amount of learning before use. Visual feedback can be provided primarily during stationary exercises in sports training or rehabilitation. One way to make visual feedback useful in wearable applications is through the use of augmented reality. Some rudimentary studies using augmented reality and virtual reality with head-mounted displays are also worth investigating [47,84,110,138], although more studies are needed in this area in the future. We believe that other, simpler modalities should be thoroughly investigated before exploring the possibilities of augmented reality. In most cases where mobility is required, both haptic [23,35,36,88,159] and auditory [24,33,37,44,105,113] feedback would be more appropriate.

A special case we have observed is the use of visual feedback in swimming [45,76,77]. This includes an indirect path BMF system where a trainer or therapist is the receiver of the feedback and passes this information to the user. In this case, visual feedback is useful, but if we want to communicate directly with a swimmer, other modalities are more appropriate. In static aquatic exercises [76], the external display can serve as useful feedback for the user. A display outside of the pool is easy to implement, but it is not a wearable unit. As wearable feedback haptic or auditory feedback can be made waterproof and thus usable during exercise. These modalities also have other advantages, as the human hearing apparatus is able to detect minute changes in frequency and amplitude. Auditory feedback could be useful for many BMF applications in the future, especially in many sports where visual feedback is not appropriate. Additionally, haptic feedback with multiple vibrotactile actuators can act on different parts of the body depending on the information we want to assert.

We also found that a number of researchers use so-called “gamification” in the development of their BMF systems [43,60,126,133,139,141,157,158], mostly to make the interaction with the user more natural or lifelike and thus increase the user’s motivation. Studies using gamification as a working method have been published mainly in recent years. Although these studies usually include interesting visual components, we do not consider this a useful component in these initial studies of BMF systems; they will, however, play an important role in future development and user motivation, as demonstrated by authors in those papers.

There are also some limitations to our review, as we were not able to cover all potential aspects of reviewed papers. We focused primarily on the technical aspects of the BMF systems presented. Nevertheless, we were unable to examine some other aspects, such as the subjects, the sports and rehabilitation process and the study procedure, study objectives, motivation, and outcomes. Since our area of expertise is electrical engineering, we focused on the technical aspects rather than the other aspects of the BMF systems. However, we acknowledge that we were not able to perform the structural study of the processing algorithms used and the operation and control of the applications by the user because there were insufficient data about them in most of the papers. This was, in a way, expected, since most of the papers were written as proof-of-concept studies for BMF rather than as technical development papers. 

The initial search yielded a wide variety of papers due to the fact that our search expression included keywords that are commonly used in other areas of study, not just in BMF. This complicated the search for relevant studies, but also minimized the possibility that some relevant studies were omitted. Our review is important because, to the best of our knowledge, it is the first review paper to include only papers with closed-loop real-time BMF systems. Other reviews in this area, while comprehensive, typically examine only one or a few parts of the BMF system, whether the sensor, actuator, processing, or the trials themselves [6,7,9,10,11,163,164,165,166]. We consider our work as complementary to these reviews. We have conducted a broad review, but have not gone into detail on each element of the BMF system, as this has already been covered by other authors. This review also does not include a thorough examination of the test methods and results presented in the reviewed papers. In most cases, these are concepts and pilot studies conducted with a limited number of subjects. We believe that an in-depth knowledge of sports and rehabilitation fields is necessary to evaluate the methods used and the results acquired. We can only follow the authors’ statement if they consider their system successful. We could not find any work that does not make this claim. They differ only in their assessment of the extent of success.

## 6. Conclusions

This review of real-time BMF systems and applications highlights many interesting studies in the last 18 years. We found 144 papers that met our search and evaluation criteria. Searching three well-known databases across their lifetimes gives us good insight into the field of BMF and the evolving cycles of the different systems. This review presents the current state of research and how new technologies and new approaches are being used to solve existing and potential future problems. Real-time BMF is a relatively new area of research, as can be seen in the studies that have been found. They mostly deal with simple movements and activities, with most of them being part of rehabilitation rather than sports. This is also true for sensors and actuators, where new types of sensors and actuators are rarely used. As experts from rehabilitation, sports, and engineering work together in this research area, some conservative thinking can be observed. Solutions that work are used in multiple studies, and new ones are used only in exceptional cases, but when they are, they usually show new potential.

We anticipate that BMF systems and applications will become an important teaching, training, and rehabilitation tool for athletes and patients in a variety of settings. We can also look forward to the development of new sensors and actuators. For example, connected clothing is one of the areas with strong research activities. We are confident that the future looks bright for real-time BMF systems and applications. The technology is improving, the size and weight of wearable devices are decreasing, and production costs are falling.

The results of our study show that the field of real-time BMF systems is rapidly expanding. Exploration of existing and new sensor technologies, processing systems, communication technologies, and feedback modalities is a necessity. New activities, use cases, and training techniques are also enabling the development of new advanced BMF systems and applications. New research in engineering, sports, and rehabilitation has shown a promising future for this field. All this proves is that BMF systems will be an even more valuable tool to improve athletes’ performance and patients’ recovery in the future.

## Figures and Tables

**Table 1:** Papers classified according to the field of study and specific physical activity.

Field of Study	Activity	*N_i_*	Papers
Sport (*N* = 33)	Balance	4	[17,18,19,20]
	Cycling	3	[21,22,23]
	Dancing	1	[24]
	Golf	4	[25,26,27,28]
	Running	11	[27,29,30,31,32,33,34,35,36,37,38]
	Jumping	1	[39]
	Rowing	2	[40,41]
	Table tennis	2	[42,43]
	Skating	1	[44]
	Swimming	1	[45]
	Skiing	2	[46,47]
	Other sport activities	2	[48,49]
Rehabilitation (*N* = 111)	Balance	19	[50,51,52,53,54,55,56,57,58,59,60,61,62,63,64,65,66,67,68]
	Cycling	1	[69]
	Posture	5	[70,71,72,73,74]
	Squat	1	[75]
	Swimming	2	[76,77]
	Gait	46	[78,79,80,81,82,83,84,85,86,87,88,89,90,91,92,93,94,95,96,97,98,99,100,101,102,103,104,105,106,107,108,109,110,111,112,113,114,115,116,117,118,119,120,121,122,123]
	Other rehabilitation activities	36	[124,125,126,127,128,129,130,131,132,133,134,135,136,137,138,139,140,141,142,143,144,145,146,147,148,149,150,151,152,153,154,155,156,157,158,159]

**Table 2:** Papers classified according to the sensors utilized.

Sensor Type	*N*	Papers
Kinematic sensor (IMU)	62	[17,21,22,24,25,28,29,32,34,35,36,37,38,42,45,46,47,49,50,51,53,54,56,58,61,62,63,64,65,69,70,72,73,76,77,84,90,91,93,94,99,101,105,108,110,111,115,119,123,125,130,136,137,138,139,144,147,149,151,152,157,158,159]
Force sensor	28	[18,20,30,31,40,48,52,55,57,59,60,66,67,82,85,86,88,92,95,98,102,104,106,114,122,124,140,143]
Pressure Sensor	20	[18,19,24,33,47,78,81,83,97,100,103,105,113,117,118,120,121,132,151,155]
Stretch sensor	2	[44,153]
Bend/angle sensors	4	[24,27,146,158]
Optical motion tracking	34	[25,26,30,39,43,55,68,71,74,75,79,86,87,89,96,104,107,109,112,116,126,127,129,132,133,134,135,141,142,148,150,156,157,160]
Camera	6	[40,46,80,128,145,154]
Electromyography	4	[86,131,138,156]
Other	9	[23,40,41,45,52,69,117,124,135]

**Table 3:** Classification of sampling frequency of sensors and systems. Some studies are not included because this information is missing.

Sampling Frequency [Hz]	*N*	Papers
<100	33	[27,39,42,44,45,48,50,58,69,71,72,76,80,88,90,91,100,101,109,110,117,119,122,128,130,136,137,143,147,148,149,156]
100	27	[20,21,25,40,49,53,54,56,61,62,63,64,65,77,79,82,87,94,96,105,112,120,123,127,129,153,159]
101–499	19	[18,22,24,29,33,41,55,57,67,75,81,86,104,111,118,140,144,146,155]
500–1000	10	[37,52,55,59,66,83,98,99,102,106]
>1000	3	[30,32,34]

**Table 4:** Wearable sensor placement on the user’s body or equipment. Papers with sensors that are not wearable are not included in the list.

Sensors Location	*N*	Papers
Full body	5	[27,76,138,149,156]
Head	3	[28,50,137]
Arm	3	[127,131,146]
Hand	7	[25,69,132,136,150,158,160]
Leg	23	[21,24,32,34,35,36,61,62,63,64,84,86,87,89,91,102,110,111,115,125,130,139,151]
Foot	29	[18,22,24,31,33,37,38,44,47,52,69,78,81,83,88,90,97,100,101,103,105,108,113,117,118,120,121,123,155]
Chest	1	[70]
Back	21	[23,53,54,56,61,62,63,64,65,72,73,74,77,84,124,130,137,146,153]
Equipment	2	[25,42]

**Table 5:** BMF system architecture.

System Architecture	*N*	Papers
Compact	35	[20,28,29,33,35,37,38,39,44,50,53,58,59,66,67,68,70,82,83,84,88,95,97,100,107,120,121,123,128,133,134,142,154,155,159]
Distributed	109	[17,18,19,21,22,23,24,25,26,27,30,31,32,34,36,40,41,42,43,45,46,47,48,49,51,52,54,55,56,57,60,61,62,63,64,65,69,71,72,73,74,75,76,77,78,79,80,81,85,86,87,89,90,91,92,93,94,96,98,99,101,102,103,104,105,106,108,109,110,111,112,113,114,115,116,117,118,119,122,124,125,126,127,129,130,131,132,135,136,137,138,139,140,141,143,144,145,146,147,148,149,150,151,152,153,156,157,158,160]

**Table 6:** BMF system or application processing device type.

Processing Device	*N*	Papers
Smart Device	21	[18,22,28,31,37,50,53,58,70,72,83,84,90,94,101,103,108,113,121,139,154]
Embedded systems	16	[24,27,33,35,47,49,82,88,97,100,105,112,118,123,155,159]
Cloud/server	8	[77,93,125,132,141,151,157,158]
Computer	90	All others

**Table 7:** Papers classified according to the feedback modality and device type used.

FB Modality	Device	*N_i_*	Papers
Visual 104	Screen	74	[20,21,25,26,30,34,39,41,42,45,46,48,52,55,57,60,61,62,63,64,65,66,67,68,69,71,74,76,78,79,80,81,82,85,86,89,95,96,98,99,102,104,106,107,109,111,114,116,117,118,119,122,124,126,127,128,132,133,134,135,140,141,142,143,144,145,149,150,152,156,157,158,160]
	Smart Phone, Tablet	18	[18,19,22,38,49,72,77,78,83,90,101,121,125,136,139,151,153,154]
	Head mounted display	10	[43,47,50,75,84,93,110,132,137,138]
	Projection	5	[32,40,87,129,148]
Auditory 43	Speakers	17	[24,26,27,31,32,39,81,89,92,98,103,113,114,116,122,127,160]
	Headphones	13	[28,37,40,44,47,56,91,105,108,115,130,146,147]
	Others	13	[33,38,83,84,94,100,101,121,126,149,153,154,155]
Haptic 25	Vibrotactile actuators	19	[17,23,35,36,41,51,53,54,58,65,70,73,88,97,112,120,123,131,159]
	Others	6	[29,59,83,100,101,109]

**Table 8:** Mode of communication used in the studies. Wireless mode is further divided into specific technologies. Some papers have not specified the communication type or technology.

Communication Mode	Specific Technology	*N*	Papers
Wired		31	[26,30,37,41,42,49,52,55,56,59,67,71,79,80,81,82,87,95,96,99,106,114,117,124,126,127,129,131,140,150,160]
Wireless	Wi-Fi	4	[50,54,78,151]
	Bluetooth	33	[20,22,23,24,25,27,28,31,36,46,47,50,53,69,71,72,83,88,90,97,101,105,108,113,117,118,121,143,149,152,153,158]
	LoRa	1	[45]
	Other		[17,19,21,29,35,38,48,51,70,75,76,84,91,92,93,94,100,102,110,111,115,119,122,125,130,135,136,137,147,157,159]

**Table 9:** Primary users of BMF system.

Main User	*N*	Papers
User	50	[19,20,22,24,25,28,29,31,33,35,37,38,42,44,47,53,60,68,70,71,83,84,88,94,97,101,108,113,118,120,121,123,125,128,132,133,134,137,139,141,142,150,151,152,153,154,157,158,159]
Expert	106	[17,18,21,23,25,26,27,30,32,33,34,36,39,40,41,43,45,46,48,49,51,52,54,55,56,57,58,59,61,62,63,64,65,66,67,69,72,73,74,75,76,77,78,79,80,81,82,85,86,87,89,90,91,92,93,95,96,97,98,99,100,101,102,103,104,105,106,107,108,109,110,111,112,114,115,116,117,119,122,124,126,127,129,130,131,132,133,134,135,136,137,138,140,141,142,143,144,145,146,147,148,149,151,155,156,160]

**Table 10:** Intended BMF application use environment.

Environment	*N*	Papers
Professional	100	[17,18,21,23,25,26,27,30,32,33,34,36,38,39,40,41,43,45,48,51,52,53,54,55,56,57,58,59,61,62,63,64,65,66,67,69,72,73,74,75,77,78,79,80,81,82,85,86,87,89,90,91,92,93,95,96,97,98,99,100,101,102,103,104,106,107,109,111,112,114,115,116,117,119,122,124,126,127,129,130,131,133,134,135,136,137,138,140,141,142,143,144,145,146,147,148,149,155,156,160]
Home	30	[20,31,33,50,53,60,68,71,83,84,88,92,94,97,100,101,125,128,132,133,134,135,137,141,142,150,151,154,157,158]
In-field	27	[19,22,24,25,28,29,35,37,42,44,46,47,49,70,76,105,108,110,113,118,120,121,123,139,152,153,159]

## Data Availability

Not applicable.
